# Dissection of Emerging Shrimp Viruses Through Scientometric Assessment: Insights into Infectious Myonecrosis Virus (IMNV) and Decapod Iridescent Virus 1 (DIV1)

**DOI:** 10.3390/v17081115

**Published:** 2025-08-13

**Authors:** Kandasamy Saravanan, Rajesh Bharathi Rathinam, Sounder Abuthagir Iburahim, Jayasimhan Praveenraj, Rajendran Kiruba-Sankar, Gokhlesh Kumar

**Affiliations:** 1 ICAR-Central Island Agricultural Research Institute, Port Blair 744105, Andaman and Nicobar Islands, India; jpr948@gmail.com (J.P.); rkirubasankar@gmail.com (R.K.-S.); 2 ICAR-Central Institute of Brackishwater Aquaculture, 75, Santhome High Road, Chennai 600028, Tamil Nadu, India; 3 ICAR-Central Institute of Fisheries Education, Panch Marg, Off Yari Road, Versova, Andheri West, Mumbai 400061, Maharashtra, India; rbharathi@cife.edu.in (R.B.R.); iburahim@cife.edu.in (S.A.I.); 4 Clinical Division of Fish Medicine, University of Veterinary Medicine, Veterinarplatz 1, 1210 Vienna, Austria; 5 ICAR-Central Marine Fisheries Research Institute, Ernakulam North P.O., Kochi 682018, Kerala, India

**Keywords:** shrimp viruses, IMNV, DIV1, scientometric assessment, aquaculture

## Abstract

Viral diseases pose significant threats to global aquaculture, particularly in shrimp farming, which has suffered substantial economic losses due to pathogens such as Infectious Myonecrosis Virus (IMNV) and Decapod Iridescent Virus 1 (DIV1). This study presents a comprehensive scientometric analysis of the research landscape, knowledge structure, and emerging trends related to these two pivotal critical shrimp viruses. Using bibliometric data extracted from the Scopus database, we evaluated publication trends, key contributing countries, institutions, authors, co-authorship networks, and keyword co-occurrence patterns. IMNV-related research demonstrated more established collaborative networks, whereas DIV1 studies have surged only recently, reflecting its status as an emerging pathogen and underscoring the urgent need for intensified research efforts. Thematic clusters reveal molecular characterization, host–pathogen interactions, and viral diagnostics as central areas of focus. This analysis identifies research hotspots, collaborative gaps, and leading contributors, offering guidance for future shrimp disease research. However, challenges persist, including limited cross-border collaboration and the underrepresentation of certain regions. Our findings offer valuable insights for researchers, funding agencies, and policymakers, highlighting the opportunities for interdisciplinary and international collaboration to mitigate the impact of these viral threats in aquaculture systems.

## 1. Introduction

The global aquaculture sector, particularly shrimp farming, continues to expand rapidly and plays a critical role in ensuring food and nutritional security for the growing global population. In 2022, whiteleg shrimp (*Penaeus vannamei*) accounted for the largest share of global production, reaching about 6.8 million tonnes, primarily driven by innovations in aquaculture technologies and rising market demand [1].

Despite these advances, the shrimp aquaculture industry has consistently faced major constraints over the past three decades, mainly due to the emergence and spread of infectious diseases, especially those of viral origin [2,3]. The World Organisation for Animal Health (WOAH) lists the key viral pathogens responsible for crustacean diseases, including White Spot Syndrome Virus (WSSV), Yellow Head Virus (YHV), Infectious Myonecrosis Virus (IMNV), Taura Syndrome Virus (TSV), Decapod Iridescent Virus 1 (DIV1), and Infectious Hypodermal and Hematopoietic Necrosis Virus (IHHNV) [4]. These viruses have caused significant shrimp mortalities, decreased productivity, and international trade restrictions in major shrimp-producing regions.

Among the WOAH-listed viruses, IMNV and DIV1 have emerged more recently as major concerns due to their virulence, expanding host ranges, and increasing geographic spread. Infectious myonecrosis (IMN) is caused by the Infectious Myonecrosis Virus (IMNV), associated with mortality rates ranging from 40% to 70% in farmed shrimp populations and has resulted in severe economic losses [2]. Initially reported in *Penaeus vannamei* in Brazil in 2002 [5,6], IMNV has since spread to many countries, including Indonesia, India, and Malaysia, largely due to the transboundary movement of farmed shrimp [7]. IMNV is a non-enveloped, double-stranded RNA virus classified under the family Totiviridae and has been reported to infect *P. vannamei*, *P. monodon*, *P. merguiensis*, and *P. esculentus* [2,7]. The disease manifests as white necrotic lesions in the skeletal muscle, and the economic losses from IMN are estimated to have exceeded USD 1 billion between 2002 and 2011 [2], primarily due to the absence of effective control and prevention strategies.

Similarly, Decapod Iridescent Virus 1 (DIV1) is an enveloped icosahedral double-stranded DNA virus belonging to the family Iridoviridae. First identified in China in 2014, initially named as Shrimp Hemocyte Iridescent Virus (SHIV) in 2017, and it was later officially renamed as DIV1 in 2018 [8,9,10]. It has been associated with mortality rates of up to 80% in cultured shrimp populations [8]. Its broad host range includes *P. vannamei*, *P. japonicus*, and *Procambarus clarkii* [8,10]. The China Fishery Statistical Yearbook (2019) indicated a decline in whiteleg shrimp production from 1.5 million tonnes in 2013 to 1.2 million tonnes in 2018, largely attributed to DIV1 outbreaks [8]. Given its pathogenicity, host diversity, and geographic spread, DIV1 is now recognized as a WOAH-listed pathogen [8].

Despite their increasing relevance, both IMNV and DIV1 remain relatively under-investigated compared to better-known viruses such as WSSV and YHV [3]. Their distinct genomic properties (RNA vs. DNA), differences in transmission dynamics, and emergence in diverse geographic contexts highlight the need for more focused research [5,8]. Several critical research gaps persist, including a lack of comprehensive epidemiological data, limited understanding of host–virus interactions, and the absence of validated, standardized diagnostics, scalable point-of-care diagnostic tests, therapeutic interventions, or prophylactic measures such as vaccines [8,10]. Given these concerns, the present study focuses on IMNV and DIV1 as representative examples of RNA and DNA viruses, respectively.

The present study aims to address these gaps by employing the scientometric methods to examine global research trends, collaborations, and thematic focus areas related to IMNV and DIV1. Scientometric analysis offers a systematic and quantitative approach to mapping the research landscape and identifying priority areas for future investigation [11,12]. By providing an evidence-based overview of the existing knowledge base, this work supports informed decision-making by the researchers, policymakers, and stakeholders and promotes targeted investment in disease management strategies. Ultimately, this study contributes to the broader understanding and mitigation of emerging viral threats such as IMNV and DIV1 infections in shrimp aquaculture.

## 2. Materials and Methods

The research outputs on IMNV and DIV1 were retrieved from the Scopus database, covering the period from 2005 to 2025 for IMNV and from 2015 to 2025 for DIV1. The following keywords were used to search the literature: ‘Infectious Myonecrosis Virus’ OR ‘IMNV’ AND ‘Shrimp’ for IMNV and ‘Decapod Iridescent Virus 1’ OR ‘DIV1’ OR ‘Shrimp Hemocyte Iridescent Virus’ OR ‘SHIV’ AND ‘Shrimp’ for DIV1. For the bibliometric analysis of IMNV and DIV1, only articles indexed in Scopus were considered. While this approach may result in fewer articles compared to broader platforms such as Google Scholar, the restriction was intentionally applied to ensure bibliometric consistency and to include only peer-reviewed, high-quality publications. This approach supports the reliability and comparability of the data used to derive meaningful inferences. Among the retrieved documents, duplicates and irrelevant publications were manually screened by reviewing the titles and abstracts. Only publications that directly addressed IMNV or DIV1 in the context of host pathology, epidemiology, molecular biology, or aquaculture impact were retained for final analysis. Various parameters such as the global landscape of IMNV and DIV1 research, publications and citation trends, status of collaboration, author contribution, and thematic evolution analysis were analyzed using bibliometric analysis packages in R Studio (1.4.1106 version).

In the bibliometric analysis conducted using R Studio (1.4.1106 version) with the Biblioshiny web interface, various statistical measures were applied to evaluate the publication trends, citation patterns, and thematic developments within the research field. Co-authorship networks and keyword co-occurrence analysis were also performed to identify collaboration patterns and emerging research topics. Furthermore, a temporal analysis was carried out to track the evolution of research themes over time, which was used to provide insights into the structure and dynamics of the scientific community. Also, the details of research efforts on these two viruses were separately differentiated.

In the Biblioshiny web interface, the default parameters were retained for many of the analyses to ensure consistency and comparability across the datasets. However, specific analyses, such as trend topics, authors’ collaboration network, and thematic evolution of the parameters, were adjusted to obtain more insightful results. These adjustments included tuning the threshold for co-authorship links, adjusting the minimum frequency for keyword co-occurrence to emphasize more prominent terms, and modifying the number of clusters in the clustering algorithm to better capture the underlying research topics. By adjusting these parameters, the analysis was able to reveal deeper insights into research collaborations, thematic trends, and the evolution of research topics, leading to more meaningful interpretations and conclusions.

## 3. Results and Discussion

### 3.1. Overview of Research on IMNV and DIV1

A comparative bibliometric analysis was conducted to evaluate the research output related to Infectious Myonecrosis Virus (IMNV) and Decapod Iridescent Virus 1 (DIV1) within the defined timeframe. The findings, summarized in Table 1, reveal notable differences in the extent and nature of scientific outputs related to these two emerging viral pathogens affecting crustacean aquaculture. Between 2005 and 2025, a total of 145 publications on IMNV were recorded. In contrast, DIV1, which emerged more recently, accounted for 248 publications between 2015 and 2025. Despite its shorter publication window, DIV1 has attracted significantly greater research attention than IMNV. This heightened focus can be attributed to DIV1’s status as a relatively new and serious threat to penaeid shrimp aquaculture, its classification as an iridovirus with a broad host range, including crayfish, penaeid shrimp, palaemonid shrimp, and mud crabs, as well as geopolitical factors, particularly the fact that China, where DIV1 is endemic, has significantly higher research output and funding capacity compared to other countries [13].

Among the IMNV-related publications, 86.21% (n = 125) were original research articles, reflecting a robust scientific engagement in areas such as pathology, diagnostics, host response, and management strategies. The remaining publications included review articles (7.59%), book chapters (4.14%), and conference papers (2.07%), indicating a well-disseminated and diversified literature base. These outputs demonstrate the cumulative efforts made toward IMNV control and highlight the maturity of the research domain [14].

Similar to IMNV, publications on DIV1 were also predominantly original research articles (86.6%), followed by review articles (10.08%) and book chapters (2.01%), showcasing significant scientific engagement in exploring DIV1’s host range, diagnostic methods, tissue-level alterations, and control strategies.

Table 2 represents the key bibliometric indicators related to citations and authorship. The average citations per document for IMNV-related publications were 26.73, indicating a substantial scholarly impact and sustained relevance in aquatic animal health research. In comparison, DIV1 publications averaged 9.55 citations per document, which aligns with its relatively recent emergence as a recognized pathogen. These values underscore the well-established scientific footprint of IMNV research, while also reflecting the nascent yet rapidly growing research attention towards DIV1. A total of 619 authors contributed to IMNV-related publications, with only four single-authored documents, highlighting a high degree of collaborative research. Similarly, 245 authors contributed to DIV1-related literature, with just 1 single-authored document. Notably, the average number of co-authors per document was higher for DIV1 (7.02) compared to IMNV (5.89), indicating a growing trend of multi-institutional and interdisciplinary collaboration, particularly in emerging disease research, which may also be influenced by the institutional structures and collaborative culture of large Chinese research consortia [15].

The collaborative nature of DIV1 research could be attributed to the urgent need for coordinated surveillance and diagnostics across national borders, particularly as DIV1 continues to pose a threat to both marine and freshwater crustaceans in Asia [16]. Additionally, increased international awareness and the inclusion of DIV1 in the list of reportable diseases by the World Organisation for Animal Health (WOAH, formerly OIE) catalyzed global research partnerships.

### 3.2. Global Research Landscape of IMNV and DIV1

**Figure 1:**
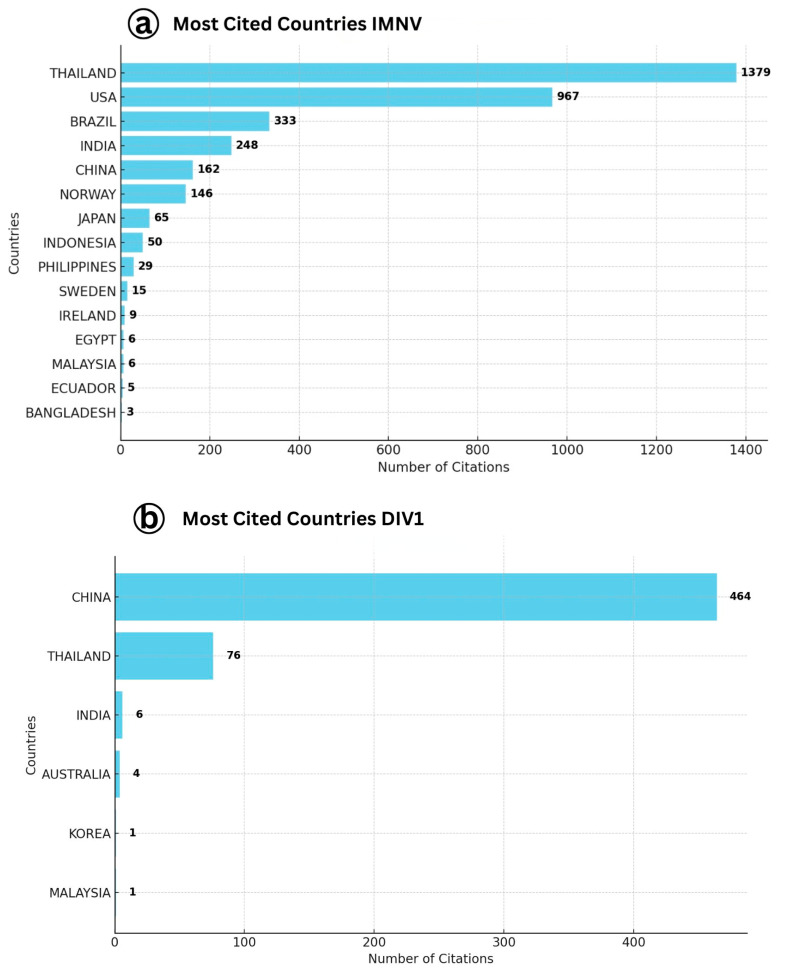
(**a**) Details showing the most cited countries with number of citations on Infectious Myonecrosis Virus (IMNV) between 2005 and 2025 (Thailand: 1379) and (**b**) Decapod Iridescent Virus 1 (DIV1) between 2015 and 2025 (China: 1230).

A detailed analysis of country-wise citation trends (Figure 1) highlights the primary contributors to the global research output on IMNV and DIV1. Among the countries, Thailand leads IMNV-related research impact with 1379 total citations, reflecting its dominant role in the field. The United States follows with 967 citations, while Brazil ranks third with 333 citations. Thailand’s prominence is likely attributed to its extensive shrimp aquaculture industry, which has faced recurrent IMNV outbreaks. These challenges have catalyzed concentrated research efforts and international collaboration, establishing Thailand as a central hub for IMNV-related studies [17]. Similarly, the USA has contributed foundational work in virology and diagnostics, while Brazil, being the country where IMNV was first reported in *P. vannamei* culture systems, has also contributed substantially to its early characterization and management [6].

In contrast, for DIV1, China was identified as the most productive and cited country, contributing 1230 citations, followed by Thailand (115 citations) and the Czech Republic (94 citations). The dominance of China in DIV1 research is unsurprising, given that the virus was initially characterized in Chinese aquaculture systems and has since posed a growing threat to local shrimp and crayfish farming [9]. The high citation count reflects both the volume and impact of China’s research on this emerging viral pathogen. The involvement of Thailand is consistent with its broader investment in aquatic animal health, while the contribution of the Czech Republic is likely driven by its interest in virological taxonomy and molecular characterization of iridoviruses in both freshwater and marine crustaceans. These findings emphasize how the emergence and spread of viral diseases in shrimp aquaculture influence national research priorities. Countries with significant shrimp farming industries tend to be at the forefront of research on emerging pathogens due to the economic implications and biosecurity concerns.

### 3.3. Author Collaboration

**Figure 2:**
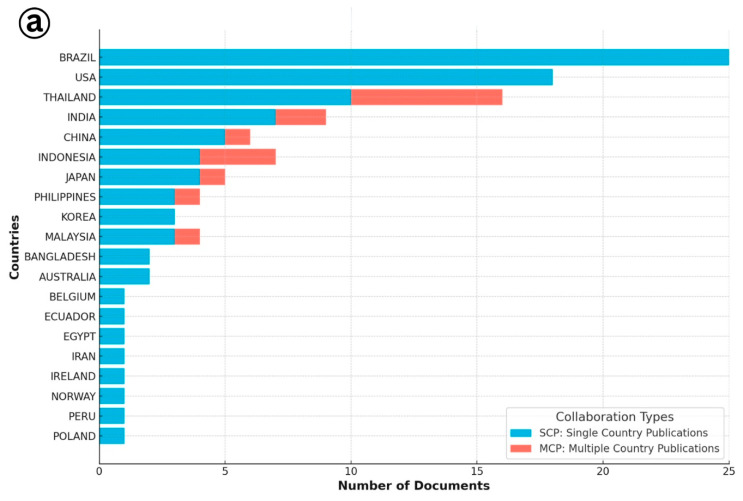

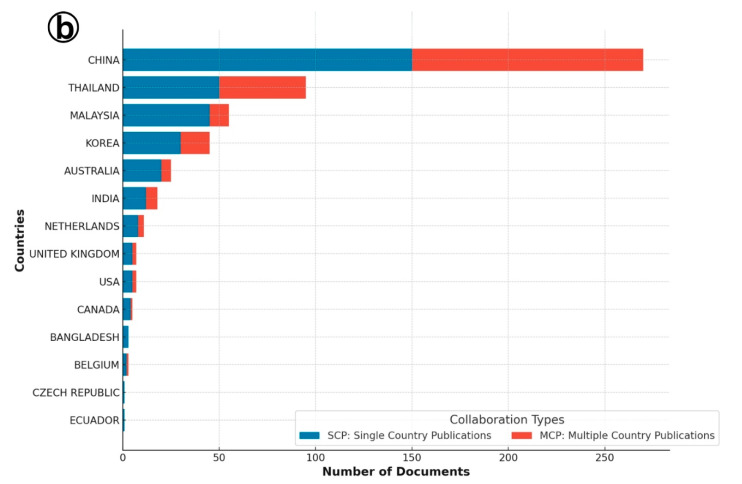
(**a**) Collaboration details of single and multiple country publications on Infectious Myonecrosis Virus (IMNV) between 2005 and 2025 and (**b**) Decapod Iridescent Virus 1 (DIV1) between 2015 and 2025.

The analysis of authorship patterns revealed important insights into the geographic distribution and collaborative trends in research on IMNV and DIV1 (Figure 2). For IMNV-related publications, Brazil emerged as the leading country, hosting the highest number of corresponding authors, followed by the United States and Thailand. This aligns with the historical significance of Brazil, as the first country to report IMNV in cultured *P. vannamei* in the early period of the year 2000 [6], and its continued research engagement in viral diagnostics and management. The USA and Thailand have also played key roles in characterizing IMNV and advancing diagnostics and surveillance tools, supported by their extensive shrimp farming industries and established aquatic health research infrastructure [17]. In terms of international collaborations, the USA led with the multi-country author affiliations, followed by Thailand and India. This highlights the globalized effort to combat IMNV through knowledge sharing and cross-border research partnerships. In addition to these countries, several countries such as Indonesia, Malaysia, Australia, Bangladesh, Belgium, Egypt, Iran, Ireland, Norway, Peru, and Poland were engaged in single-country publications, reflecting their localized research contributions and perhaps region-specific disease challenges or resource capabilities.

In contrast, the authorship analysis for DIV1 publications revealed a more centralized pattern. China stood out as the leading country in terms of both single-country and multi-country collaborations. This is consistent with its foundational role in DIV1 research, as the virus was first reported and characterized in China [9], and the country continues to lead ongoing investigations. Thailand and Malaysia also ranked among the top corresponding author countries, indicating rising regional attention to this emerging viral threat. Single-country publications on DIV1 were also reported from India, Indonesia, Canada, and Ecuador, highlighting the global spread and research interest in DIV1. Collectively, these findings suggest that while IMNV research reflects an established and widely collaborative scientific network, DIV1 research is in a developing phase marked by strong regional leadership and expanding global engagement.

**Figure 3:**
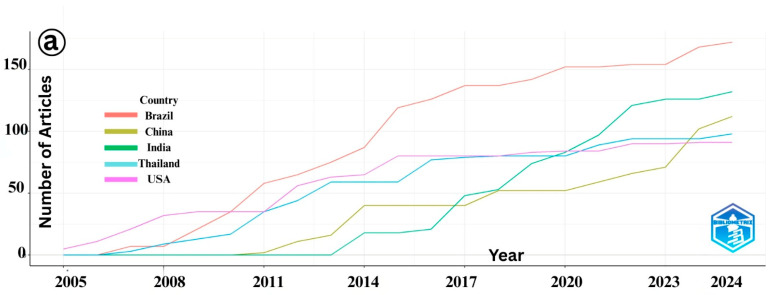

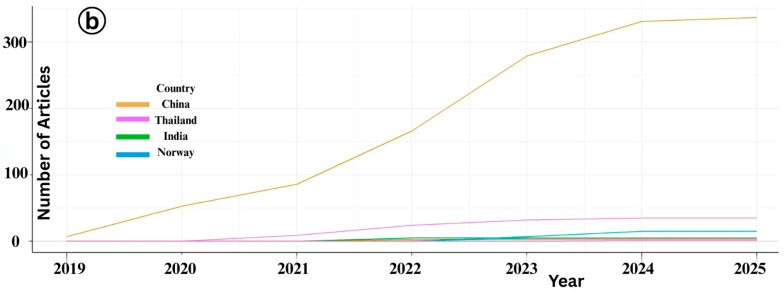
(**a**) Country-wise number of publications published over time on Infectious Myonecrosis Virus (IMNV) between 2005 and 2025 and (**b**) Decapod Iridescent Virus 1 (DIV1) between 2015 and 2025.

An analysis of the publication trends on IMNV reveals a general increase in scientific output from the top five contributing countries, such as Brazil, China, India, Thailand, and USA, reflecting their active engagement in addressing this viral threat to shrimp aquaculture [18] (Figure 3a). However, a plateau in publication output was observed in India and Thailand after 2016, indicating a potential shift in research priorities or changes in resource allocation within these countries. In contrast, research on DIV1 shows a marked and continuous rise in publications, predominantly driven by China (Figure 3b). Since DIV1 was first identified in Chinese aquaculture systems, the country has maintained it. This sustained effort is likely due to the substantial economic impact of DIV1 on China’s shrimp and crayfish farming industries, which has prompted extensive studies aimed at understanding and mitigating the virus’s effects.

### 3.4. Publication and Citation Trends: A Comparative Analysis for IMNV and DIV1

**Figure 4:**
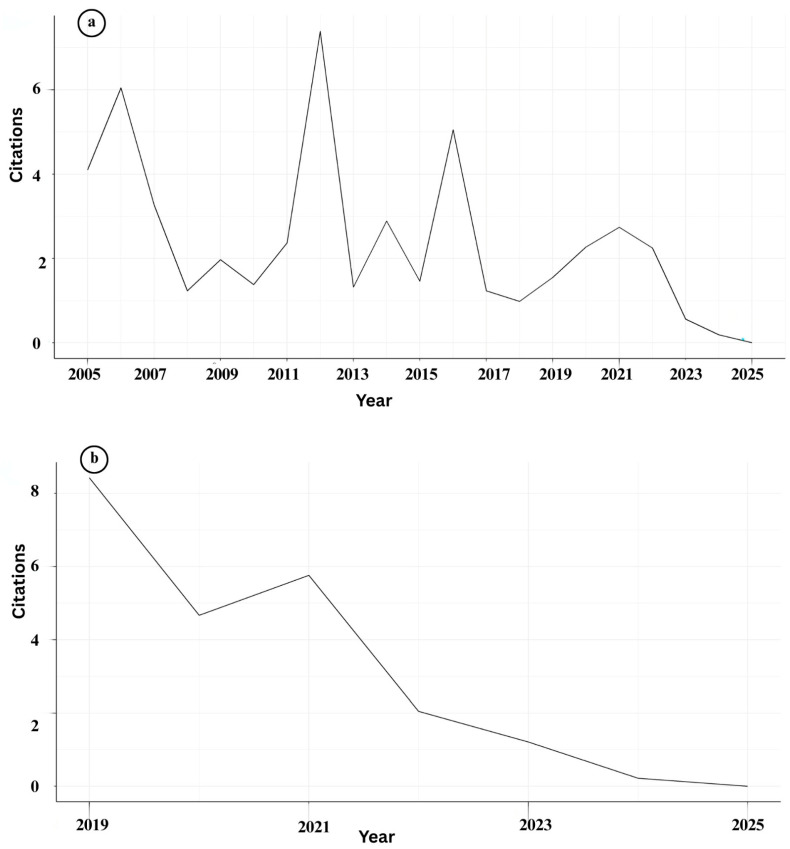
(**a**) Details of year-wise citations of publications on Infectious Myonecrosis Virus (IMNV) between 2005 and 2025 and (**b**) Decapod Iridescent Virus 1 (DIV1) between 2015 and 2025.

The bibliometric evaluation of IMNV research reveals fluctuating trends in both publication output and citation impact over the past two decades (Figure 4a and Table 3). The Average Citation Per Publication (ACPP) peaked in 2012 for IMNV, with another significant increase in 2016, indicating the release of influential studies or heightened scholarly attention during these periods [17]. The highest number of IMNV-related publications was recorded in 2024, suggesting a recent resurgence in research interest, which was potentially driven by the emergence of novel IMNV variants reported in Brazil [18]. However, notable declines in publication numbers were recorded in 2010, 2013, 2018, and 2023, indicating intermittent research engagement within the research community. These fluctuations suggest that while IMNV continues to be a relevant research topic, it is periodically overshadowed by emergent pathogens or evolving priorities in aquaculture health management.

In contrast to IMNV, research on DIV1 showed a linear and consistent increase in publication output since 2015, highlighting its rising prominence as a subject of scientific investigation (Figure 4b and Table 3). The ACPP for DIV1 peaked in 2018, likely reflecting the initial period of international recognition and concern following the first characterization of the virus in China [9]. The highest number of DIV1-related publications was recorded in 2023, while the peak citations occurred for articles published in 2022. This trend suggests not only a quantitative rise in research output but also an improvement in the quality and relevance of recent studies. The higher citation counts in 2022 compared to 2021 indicate that newer research is gaining traction and visibility, likely due to better characterization methods, growing global awareness, and strengthened international collaboration.

### 3.5. Author Contribution to IMNV and DIV1 Research

**Figure 5:**
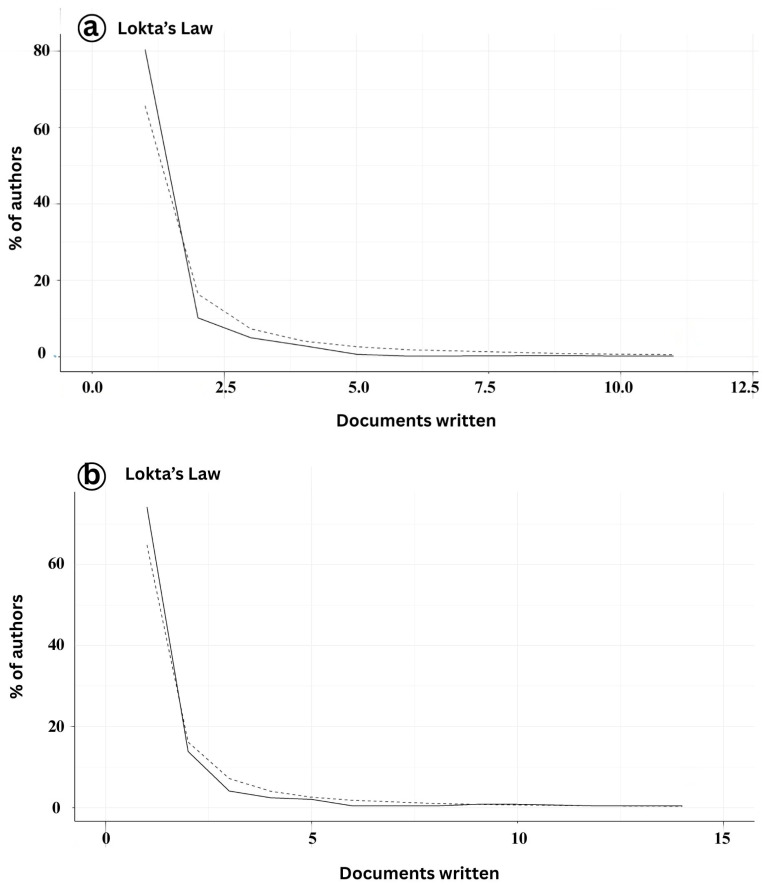
(**a**) Author productivity of publications on Infectious Myonecrosis Virus (IMNV) between 2005 and 2025 and (**b**) Decapod Iridescent Virus 1 (DIV1) between 2015 and 2025.

The analysis of author productivity in the domains of Infectious Myonecrosis Virus (IMNV) and Decapod Iridescent Virus 1 (DIV1) research aligns with the patterns described by Lotka’s Law [19] (Figure 5a). Lotka’s Law is a statistical principle that describes the distribution of scientific productivity, particularly in terms of publications. Our findings showed that the distribution of scientific productivity on IMNV was significantly skewed, as most authors are at the low end of the distribution, with only a few at the high end. This distribution is characteristic of mature or well-established research domains, where a core group of researchers drives ongoing scientific progress and maintains the field’s development over time [20].

In comparison, author productivity on DIV1 research was notably lower, with 60% of contributors authoring fewer than five publications during the study period (Figure 5b). This pattern likely reflects the relatively recent emergence of DIV1 as a significant pathogen in aquaculture, with research efforts still dispersed across a broader base of generalists or early contributors [9]. Such trends are common in emerging disease research, where initial investigations are conducted by a wide range of researchers exploring the problem from diverse disciplinary and regional perspectives.

**Figure 6:**
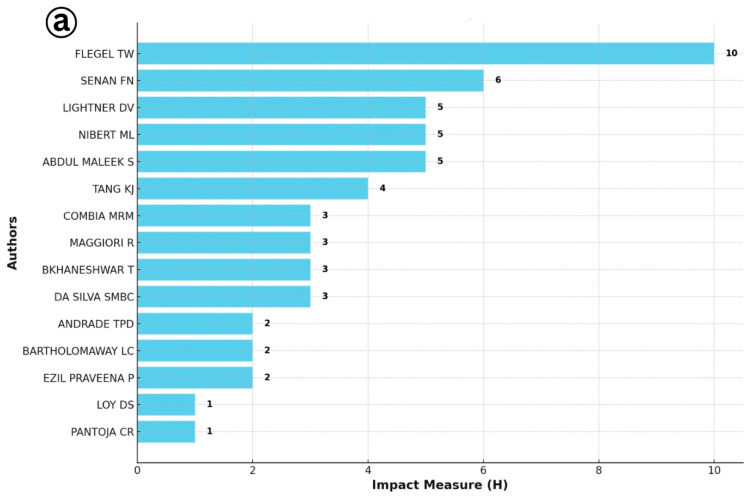

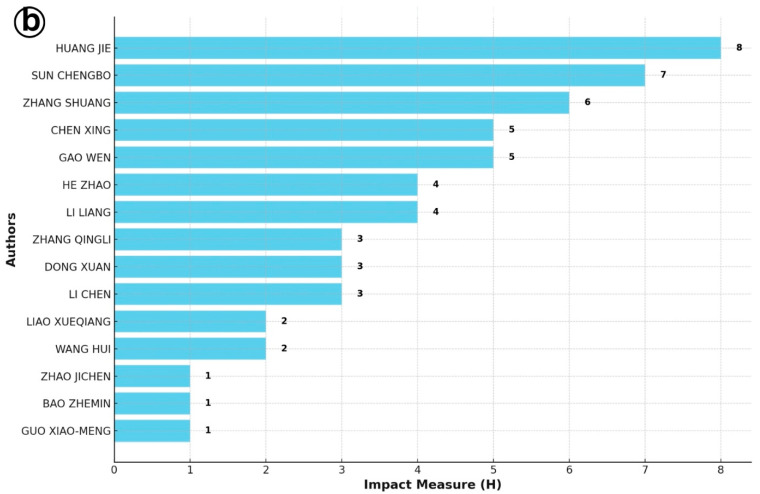
(**a**) Most productive author analysis for Infectious Myonecrosis Virus (IMNV) between 2005 and 2025 and (**b**) Decapod Iridescent Virus 1 (DIV1) between 2015 and 2025.

The identification of the most productive authors based on the h-index offers critical insight into the intellectual leadership and influence within a specialized research domain (Figure 6). In the context of IMNV research, the h-index analysis highlighted Flegel T.W., Senapin S., and Lightner D.V. as the most prolific and influential contributors. These researchers have made significant contributions to the understanding of IMNV, particularly in the areas of molecular biology, epidemiology, and pathology of IMNV in penaeid shrimp. Their work has been especially impactful in countries severely affected by IMNV outbreaks, where their research has supported the efforts on disease management and aquaculture sustainability [14,17,21].

Similarly, in the case of DIV1, an emerging pathogen of concern in shrimp and crayfish aquaculture, the h-index-based analysis revealed Huang Jie, associated with the Network of Aquaculture Centres in Asia-Pacific (NACA), as the leading contributor. He was followed by Sun Chengbo and Shuang Zhang from Guangdong Ocean University, China. These researchers have led several important studies focusing on the early detection, molecular characterization, and pathogenicity of DIV1, particularly in the Chinese aquaculture sector, where the virus has caused significant concern since its first report [9].

**Figure 7:**
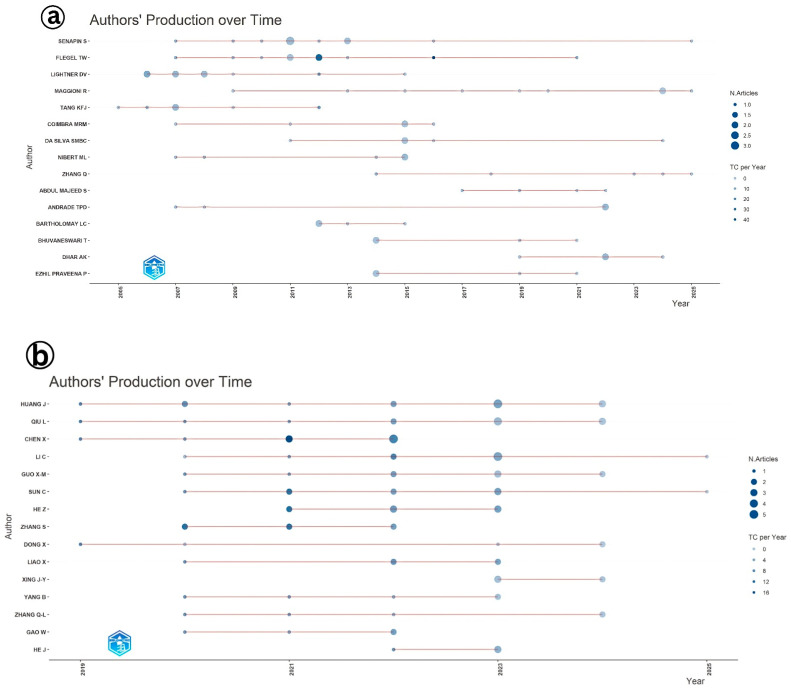
(**a**) Authors’ production over time of the scientific articles published on Infectious Myonecrosis Virus (IMNV) between 2005 and 2025 and (**b**) Decapod Iridescent Virus 1 (DIV1) between 2015 and 2025.

An analysis of authors’ productivity over time provides insights into the evolution and continuity of research interest in viral diseases affecting shrimp aquaculture (Figure 7). In the case of IMNV, researchers such as Senapin S., Flegel T.W., and Lightner D.V. demonstrated consistent contributions to the scientific literature across several years, reflecting their sustained engagement and leadership in the field. The visual representation, where circle size denotes the research productivity, further revealed that Flegel T.W. not only published frequently but also received a higher number of citations, suggesting the significance and impact of his contributions to the global shrimp health research community [14,17,21]. The consistent publication activity across various authors indicates a stable and ongoing research commitment to the understanding and management of IMNV. In contrast, for DIV1, a relatively newer viral threat, the analysis of author productivity showed a distinct upward trend, with the maximum number of publications recorded in 2023. This surge reflects the growing global attention toward DIV1, which has emerged as a serious pathogen impacting farmed shrimp and crayfish [9].

### 3.6. Collaboration Patterns in IMNV and DIV1 Research

**Figure 8:**
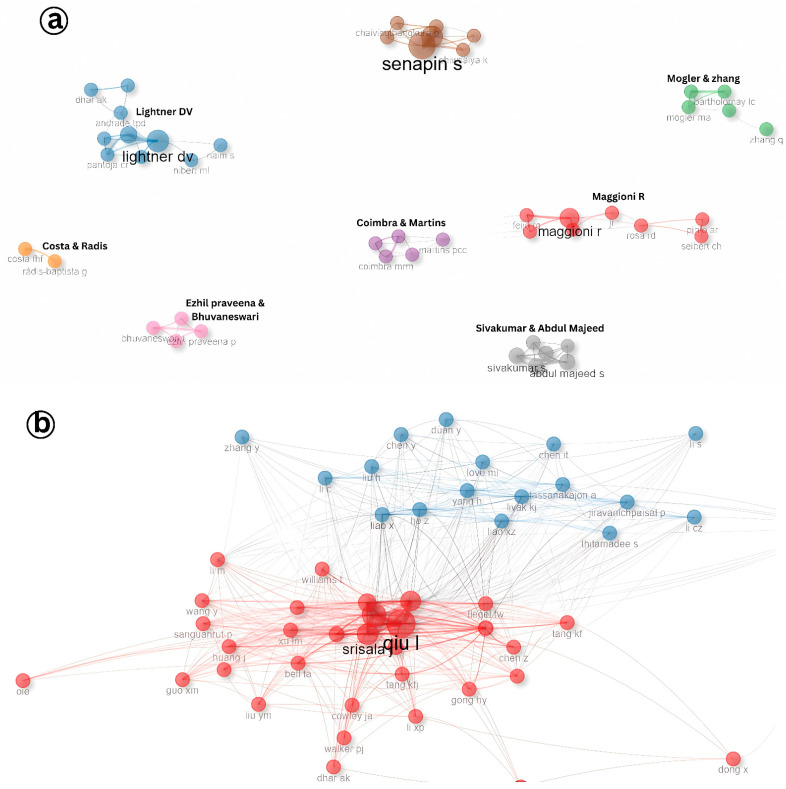
(**a**) Collaboration pattern of author research on Infectious Myonecrosis Virus (IMNV) between 2005 and 2025 and (**b**) Decapod Iridescent Virus 1 (DIV1) between 2015 and 2025.

Collaboration among researchers plays a pivotal role in advancing scientific understanding, especially in specialized fields such as aquatic animal virology. The analysis of author collaboration patterns in Infectious Myonecrosis Virus (IMNV) research identified eight prominent research clusters led by Senapin, Lightner, Ezhil Praveena, Abdul Majeed, Maggioni, Radis-Baptista, Martins, and Zhang (Figure 8a). These authors have made significant contributions to the global understanding of IMNV, a major viral pathogen in shrimp aquaculture. Notably, Dr. Lightner and his collaborators at the University of Arizona have been instrumental in the early identification and histopathological characterization of IMNV, laying the foundation for subsequent epidemiological and molecular studies [14].

In contrast, the collaboration landscape for DIV1 research appears more centralized, characterized by two dominant research clusters led by Srisala and Qiu. The close partnership among these groups indicates a focused yet expanding research community (Figure 8b). Given that DIV1 is a relatively recent addition to the spectrum of shrimp viral pathogens, first reported in China around 2014 [9], the current collaboration patterns reflect the early-stage consolidation of knowledge and expertise in the field.

**Figure 9:**
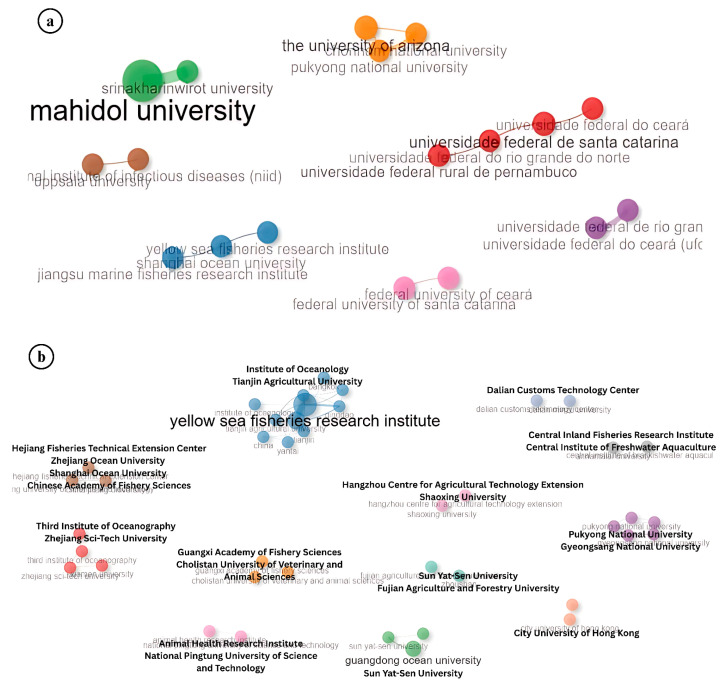
(**a**) Collaboration pattern of institutions in research on Infectious Myonecrosis Virus (IMNV) between 2005 and 2025 and (**b**) Decapod Iridescent Virus 1 (DIV1) between 2015 and 2025.

The analysis of institutional collaborations in IMNV research revealed both international and domestic partnership trends (Figure 9a). Notably, strong collaborative linkages between South Korea and the USA, as well as India and Sweden, demonstrate the international dimension of IMNV research. These partnerships often stem from bilateral projects, student exchange programs, and co-authorship publications in peer-reviewed journals [22]. Conversely, institutions in China, Brazil, and Thailand were found to engage primarily in intra-national collaborations. This pattern may reflect the availability of robust national research funding, domestic shrimp aquaculture priorities, and institutional research capacities within these countries [21].

A similar trend of national-level institutional collaboration was observed in DIV1 research, particularly among Chinese universities and research centres, reflecting China’s leadership in initial detection and characterization of this emerging viral pathogen (Figure 9b) [9]. However, the collaboration between Guangdong Ocean University and Pukyong National University in South Korea stands out as an example of transnational institutional collaboration in DIV1 research. This partnership likely stems from regional concerns over disease transmission and the need for shared surveillance efforts across East Asia—a region with high shrimp production and frequent transboundary movement of live aquatic animals [2].

### 3.7. Journal Impact on IMNV and DIV1 Research

**Figure 10:**
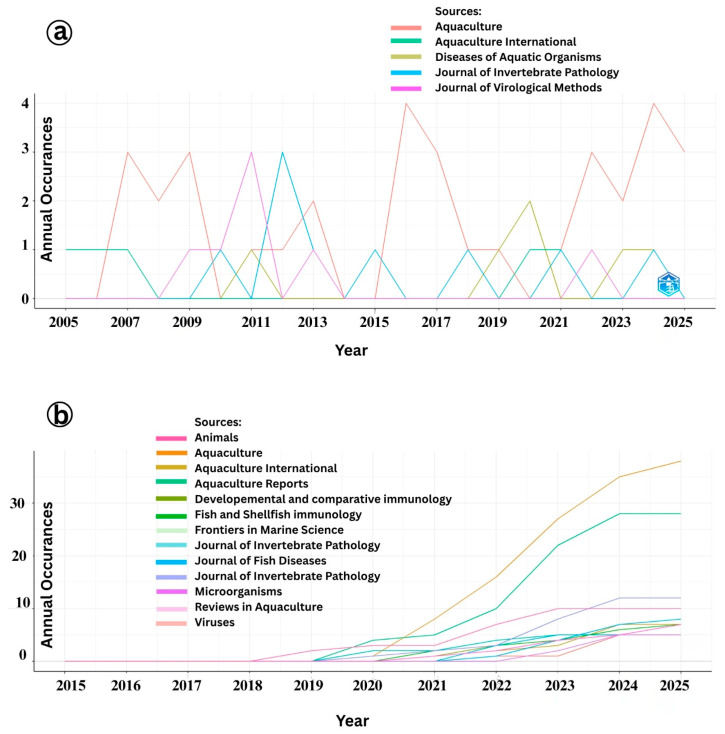
(**a**) Impact of journals in research on Infectious Myonecrosis Virus (IMNV) between 2005 and 2025 and (**b**) Decapod Iridescent Virus 1 (DIV1) between 2015 and 2025.

The temporal trends in journal-wise publication analysis on Infectious Myonecrosis Virus (IMNV) and Decapod Iridescent Virus 1 (DIV1) offer insights into the evolving scientific interest and response to disease outbreaks in shrimp aquaculture (Figure 10a). The peak in IMNV-related publications in the Aquaculture journal in 2006, 2015, and 2021 corresponds to key periods of the virus’s initial emergence, spread, and resurgence in shrimp farming regions. The consistent publication of IMNV-related reports in the journal Diseases of Aquatic Organisms, known for its focus on first reports and incidence of aquatic diseases, further supports the pattern of IMNV being a recurring and regionally significant pathogen. Moreover, the gradual rise in IMNV-related publications in the Journal of Virological Methods from 2008 to 2013 and in the Journal of Invertebrate Pathology post-2009 highlights the structured progression of research from initial identification and outbreak characterization to the development of diagnostic methodologies and detailed pathological investigations [2,23].

In contrast, DIV1-related publications began gaining momentum in Aquaculture International and Developmental and Comparative Immunology only after 2019, continuing through 2024 (Figure 10b). This trend correlates with the emergence and recognition of DIV1 as a novel and significant viral pathogen in shrimp and other crustaceans since its first formal description in China [9]. The notable rise in publications in Developmental and Comparative Immunology reflects a growing focus on understanding the host immune response and pathogenesis of DIV1, an essential aspect in developing effective management and therapeutic strategies. Similarly, the increased publication frequency in the Journal of Invertebrate Pathology during the same period reflects a deepening interest in elucidating the histopathology and tissue tropism of DIV1 infections, consistent with the standard progression of investigative focus after pathogen emergence.

**Figure 11:**
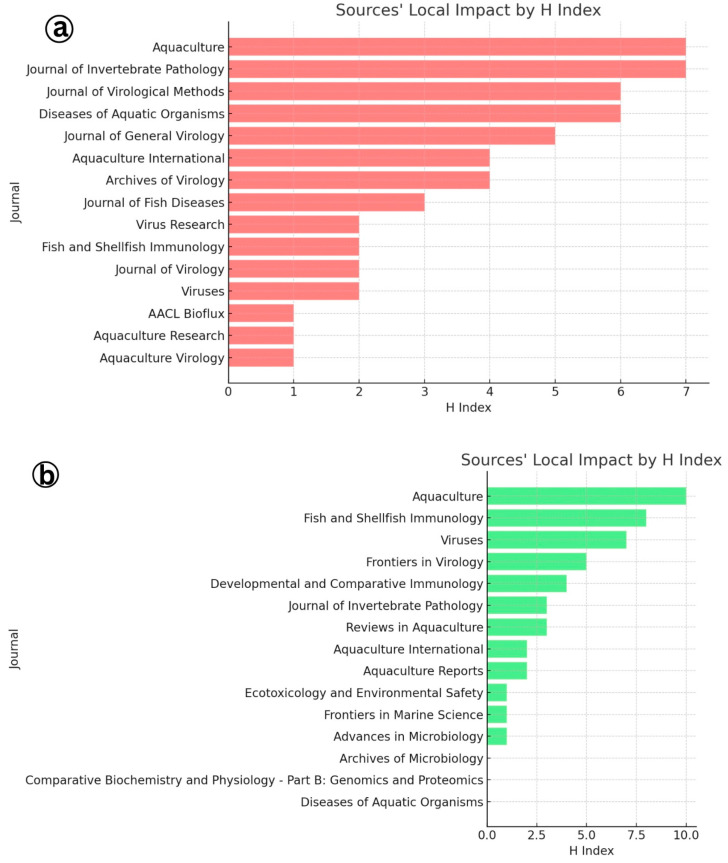
(**a**) Local impact analysis of journals in research on Infectious Myonecrosis Virus (IMNV) between 2005 and 2025 and (**b**) Decapod Iridescent Virus 1 (DIV1) between 2015 and 2025.

**Figure 12:**
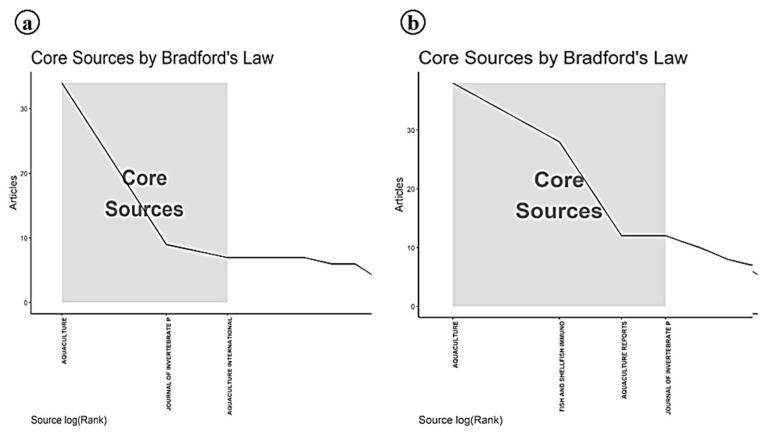
(**a**) Bradford’s law analysis of journals in research on Infectious Myonecrosis Virus (IMNV) between 2005 and 2025 and (**b**) Decapod Iridescent Virus 1 (DIV1) between 2015 and 2025.

The identification of Aquaculture and the Journal of Invertebrate Pathology as the most productive journals for publishing research on Infectious Myonecrosis Virus (IMNV), as revealed through local impact analysis and Bradford’s Law, which emphasizes their central role in disseminating aquaculture-related scientific knowledge (Figure 11 and Figure 12). These journals consistently attracted high-quality submissions on IMNV, reflecting their strong thematic alignment with virological, pathological, and applied aquaculture research. Aquaculture has long been recognized for its broad coverage across disciplinary, interdisciplinary, and transdisciplinary themes in aquaculture, making it a natural outlet for studies addressing emerging diseases like IMNV. Similarly, the Journal of Invertebrate Pathology serves as a specialized platform for detailed pathogenetic and histopathological investigations, which are essential for the accurate characterization of novel viral infections in shrimp and other crustaceans.

In this study, Aquaculture and Fish and Shellfish Immunology journals were further identified within this core group, highlighting their pivotal roles in publishing immunological, diagnostic, and disease management strategies related to DIV1. The latter journal, Fish and Shellfish Immunology, particularly focuses on host immune responses and the development of novel prophylactic measures, aligning with the increasing need for understanding host–pathogen interactions in the face of emerging viral threats [24].

### 3.8. Temporal Evolution of IMNV and DIV1 Research

**Figure 13:**
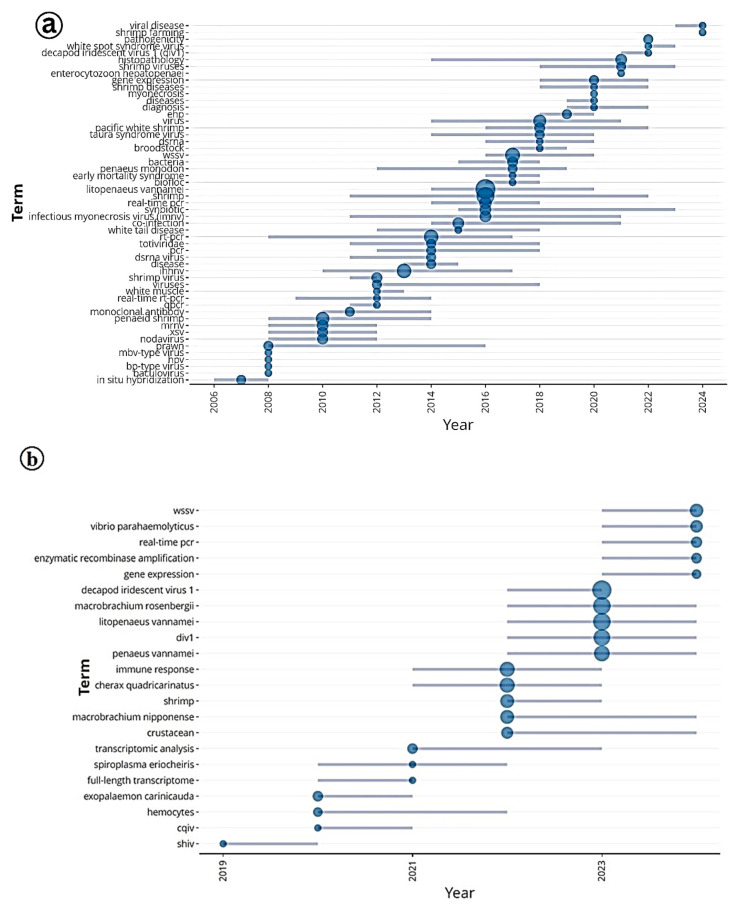
(**a**) Trend topic analysis of research on Infectious Myonecrosis Virus (IMNV) between 2005 and 2025 and (**b**) Decapod Iridescent Virus 1 (DIV1) between 2015 and 2025.

The trend topics analysis provides valuable insights into the evolving research landscape concerning Infectious Myonecrosis Virus (IMNV) and Decapod Iridescent Virus 1 (DIV1) in shrimp aquaculture from 2005 to 2024 (Figure 13). In the early years, the prominence of terms such as in situ hybridization, alongside transient keywords such as baculovirus, nodavirus, MBV-type virus, HPV, and BP-type virus, highlights the initial efforts toward viral identification and classification.

These findings align with the historical context of IMNV’s emergence in Brazil in 2002 and its subsequent global spread, prompting extensive diagnostic exploration across various viral families [14,23]. Between 2012 and 2020, the frequent use of the keyword Biofloc reflects a notable shift in research focus toward sustainable and resilient shrimp farming practices. Biofloc technology (BFT) gained prominence during this period for its ability to improve water quality, facilitate nutrient recycling, and potentially enhance shrimp resistance to viral infections such as IMNV. From 2016 onwards, the emergence of keywords related to co-infections such as EHP (*Ecytonucleospora hepatopenaei*), WSSV (White Spot Syndrome Virus), DIV1, TSV (Taura Syndrome Virus), and EMS (Early Mortality Syndrome) indicates a growing recognition of the complex etiological challenges in shrimp aquaculture. Research has shown that co-infections can exacerbate disease severity and complicate diagnostics, highlighting the need for integrated surveillance and comprehensive health management strategies [2].

The trend analysis for DIV1, particularly from 2019 onwards, reveals a change in research attention, coinciding with the virus’s first reports in China and its subsequent detections across Asia. The prominence of WSSV, *Vibrio parahaemolyticus*, real-time PCR, and Decapod Iridescent Virus 1, among the frequent terms, suggests a focus on rapid diagnostics and pathogen interactions, which are critical in mitigating disease outbreaks in intensive farming systems [9]. The emergence of new topics such as SHIV (possibly a misidentified abbreviation or referring to a novel virus) and *Exopalaemon carinicauda* (a non-Penaeid shrimp species) from 2021 indicates an expanding scope of research into host range and the identification of novel pathogens in aquaculture, which could represent the next frontier in shrimp disease studies.

Additionally, the sustained presence of species-specific terms like *Penaeus vannamei* and *Litopenaeus vannamei* reflects the continued dominance of this species in global shrimp aquaculture and its central role in most virological research. Meanwhile, the recurrence of keywords, such as gene expression, immune response, and transcriptomic analysis, denotes long-standing and increasingly sophisticated efforts to understand host–pathogen interactions at the molecular level. These insights are paving the way for the development of targeted disease interventions and selective breeding programs aimed at enhancing disease resistance.

Thematic analysis was performed with centrality relevance as the core criterion. The resulting plot was divided into four distinct themes through both vertical and horizontal analysis. Within the niche themes of IMNV research, keywords such as brood stock, myonecrosis, LAMP, dsRNA virus, and phylogenetic analysis emerged. These themes represent emerging or specialized areas in shrimp farming that, while not yet central to the current focus, hold significant potential for future exploration. The rapid evolution of molecular-based detection assays and the growing utilization of RNAi technology for shrimp disease diagnostics and control likely contribute to the prominence of these findings [25,26].

**Figure 14:**
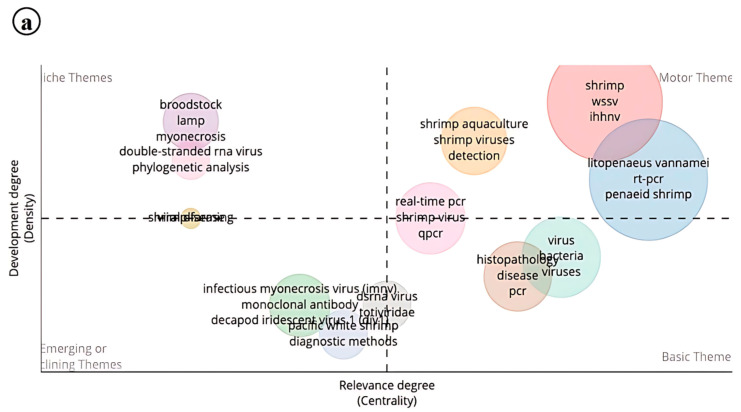

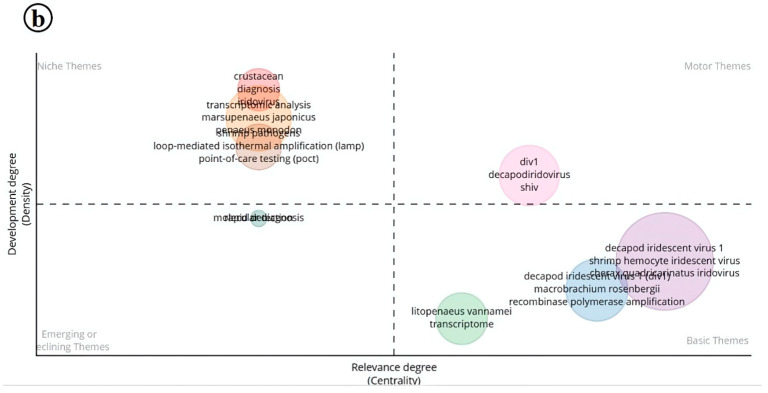
(**a**) Thematic analysis of research on Infectious Myonecrosis Virus (IMNV) between 2005 and 2025 and (**b**) Decapod Iridescent Virus 1 (DIV1) between 2015 and 2025.

In the motor themes, keywords such as shrimp aquaculture, viruses, detection of WSSV, IHHNV, and real-time PCR reflect a high level of active research development in core areas of IMNV research. Notably, viral interference between WSSV and IMNV has been documented with the help of qPCR techniques [27]. The basic themes include PCR and histopathology, underscoring the continued relevance of these foundational techniques for the accurate identification of IMNV in shrimp farming. Meanwhile, in the emerging field, monoclonal antibodies, DIV1, and IMNV show declining research efforts in those research topics. However, molecular diagnostic assays continue to play a vital role in preventing disease outbreaks, thereby safeguarding the sustainability and productivity of shrimp farming [28]. The availability of monoclonal antibodies for IMNV and the widespread use of molecular detection techniques may explain the current classification in the emerging field (Figure 14a).

In DIV1 research, niche themes include keywords such as “Transcriptomic analysis”, “LAMP”, and “Point-of-care testing”. Although these themes currently show low centrality, they exhibit significant potential for future development. Notably, both the motor and emerging themes lack substantial representation, suggesting that key transformative areas in DIV1 research are still underexplored. On the other hand, the basic theme is characterized by keywords like recombinase polymerase amplification (RPA) and transcriptome, pointing to the fundamental and preliminary stages of research in this area. These themes indicate that the current focus of DIV1 research is on transcriptomic-level studies, which are advancing rapidly, particularly in the context of diagnostics and pathogen identification. RPA is used for the rapid detection of DIV1, and further advancements like real-time RPA have also been developed for this pathogen [29] (Figure 14b).

**Figure 15:**
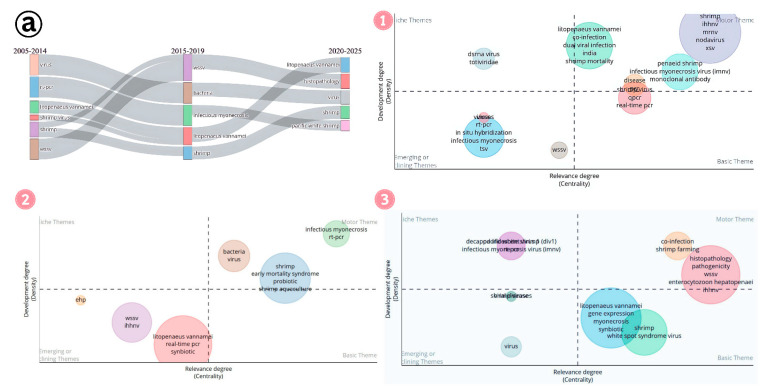

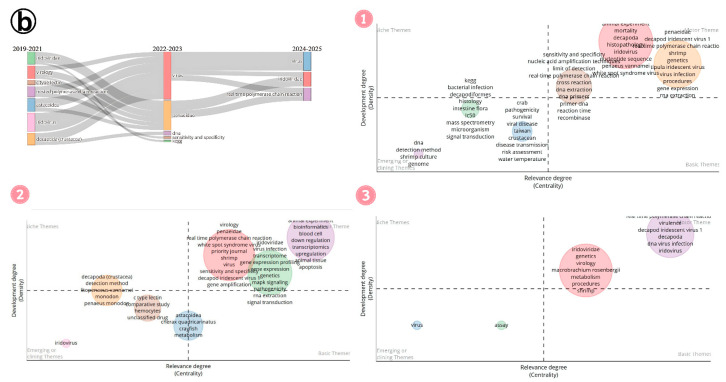
(**a**) Thematic evolution of research on Infectious Myonecrosis Virus (IMNV) between 2005 and 2025: (1) Initial phase (2005–2014). (2) Mid phase (2015–2019). (3) Latest phase (2020–2025). (**b**) Thematic evolution of research on Decapod Iridescent Virus 1 (DIV1) between 2019 and 2025: (1) Initial phase (2019–2021). (2) Mid phase (2022–2023). (3) Latest phase (2024–2025).

Thematic evolution helps us to understand the research landscape of IMNV studies from 2005 to 2025 (Figure 15a). In the initial phase of research, detection and co-infection with different virus and bacterial pathogens gained momentum. During the middle stage, keywords such as probiotic, early mortality syndrome, and synbiotics gained prominence, reflecting a change in research focus from mere detection to control strategies in shrimp farming. In the most recent period (2020–2025), frequently used keywords include co-infection, synbiotics, and both advanced and conventional diagnostic techniques, indicating a continued emphasis on diagnostics and integrated disease management. Meanwhile, within the basic themes, studies on gene expression emerged, highlighting foundational efforts to understand host–pathogen interactions. The appearance of the keyword pathogenicity within the motor themes further emphasizes the growing importance of investigating virulence factors at the genomic level, marking a significant step towards understanding the molecular mechanisms of IMNV.

Between 2019 and 2025, research on DIV1 in shrimp farming evolved significantly (Figure 15b). Initially, foundational themes like host and pathogen identification dominated the field, representing well-established research areas that were crucial for understanding the disease or its causative agent. As the field progressed, new and emerging themes like SHIV and CQIV gained prominence, reflecting a growing focus on novel viruses and advanced diagnostic methods. In parallel, the research expanded to species-specific studies while still in the emerging phase, indicating a rising interest in understanding the genetic and immune response of economically important shrimp species against this pathogen. During the mid-phase of research, efforts shifted toward examining host range, developing diagnostic tools, and investigating immune responses. Over time, this focus was gradually shifted towards omics-based approaches such as transcriptomics and various bioinformatics analyses. While traditional areas like pathogen characterization, host biology, and farming systems remain central to shrimp farming research, new themes have begun to emerge. These include co-infection with *Vibrio parahaemolyticus*, the genetic diversity of the pathogen, and detailed host immune responses. These emerging areas signal a broader and deeper exploration of shrimp health and disease resistance. As researchers continue to investigate shrimp biology, these under-explored themes hold significant potential for future advancements in the field.

Simultaneously, the growing attention to environmental factors and species-specific pathology reflects a change toward examining broader ecological and environmental influences on shrimp health. This trend suggests that future research may move beyond traditional concerns to encompass a wider range of environmental and ecosystem-level factors. Such developments highlight the dynamic nature of DIV1 research in shrimp farming, which continues to adapt to emerging challenges, technologies, and areas of inquiry. Meanwhile, growth performance and immunity continued to be at the center, indicating the constant focus on improving shrimp farming productivity and disease management. In contrast, interest in rapid detection methods and co-infections with other viruses, such as white spot syndrome virus, has declined in recent years as newer and more specialized topics gained prominence. This evolving research landscape reveals a dynamic shift from conventional topics to more focused and advanced areas of study from 2019 till now, demonstrating the adaptability and forward-thinking approach of DIV1 research in addressing the complex challenges of modern shrimp farming.

## 4. Conclusions

This scientometric analysis offers a comprehensive overview of research trends, publication patterns, and thematic focus areas related to Infectious Myonecrosis Virus (IMNV) and Decapod Iridescent Virus 1 (DIV1) over the past two decades. It identifies the key contributors, countries, and journals that have significantly shaped the scientific discourse on these economically important crustacean pathogens. While interest in these viruses has grown, the findings underscore the need for more targeted and coordinated research efforts. Specifically, future investigations should prioritize the development and validation of standardized diagnostic tools and surveillance protocols, particularly for emerging pathogens such as DIV1. There remains a critical gap in understanding cross-species transmission dynamics, host–pathogen interactions, and the ecological drivers of viral emergence. Expanding comparative virology studies can provide deeper insights into host susceptibility patterns and potential reservoirs. Moreover, fostering interdisciplinary collaboration and promoting meta-analyses to synthesize region and species-specific data will be essential to advance integrated disease management strategies. The scientometric framework employed in this study, drawing on peer-reviewed, Scopus-indexed literature and structured keyword analysis, also presents a replicable model for evaluating the research landscapes of other commercially important aquatic pathogens, such as Tilapia Lake Virus (TiLV), *Ecytonucleospora hepatopenaei* (EHP), Epizootic Ulcerative Syndrome (EUS), and Spring Viremia of Carp Virus (SVCV). Ultimately, this approach can support data-driven research prioritization, enhance global cooperation, and inform the strategic planning of aquatic animal health initiatives at national and international levels. 

## Figures and Tables

**Table 1:** Summary of literature on Infectious Myonecrosis Virus (IMNV) between 2005 and 2025 and Decapod Iridescent Virus 1 (DIV1) between 2015 and 2025.

Description	IMNV		DIV1	
	Numbers	Percentage	Numbers	Percentage
Total publications	145	100	248	100
Research papers	125	86.21	215	86.6
**Non-Peer Reviewed**				
Review	11	7.59	25	10.08
Book chapter	6	4.14	5	2.01
Conference paper	3	2.07	-	-
Book	-	-	1	0.40
Others	-	-	2	0.80

**Table 2:** Details of research publications on Infectious Myonecrosis Virus (IMNV) between 2005 and 2025 and Decapod Iridescent Virus 1 (DIV1) between 2015 and 2025.

Description	IMNV	DIV1
Average citations per document	26.73	9.55
Authors	619	245
Authors of single-authored documents	4	1
Co-authors per document	5.89	7.02

**Table 3:** Year-wise publications and citations on Infectious Myonecrosis Virus (IMNV) between 2005 and 2025 and Decapod Iridescent Virus 1 (DIV1) between 2015 and 2025.

Year	IMNV		DIV1	
	No. of Publications	Citations	No. of Publications	Citations
2005	1	86	-	-
2006	2	242	-	-
2007	5	311	-	-
2008	5	111	-	-
2009	4	134	-	-
2009	4	134	-	-
2010	3	66	-	-
2011	10	356	-	-
2012	9	931	-	-
2013	6	103	-	-
2014	11	381	-	-
2015	10	161	1	5
2016	10	505	-	-
2017	10	111	-	-
2018	5	39	-	-
2019	6	65	2	140
2020	5	68	15	299
2021	7	96	24	406
2022	10	90	56	487
2023	9	15	73	380
2024	13	5	66	93
2025	4	0	11	1

## Data Availability

All the data are provided within the article.
